# The acute effects of the thermogenic supplement Meltdown on energy expenditure, fat oxidation, and hemodynamic responses in young, healthy males

**DOI:** 10.1186/1550-2783-5-23

**Published:** 2008-12-16

**Authors:** Jean Jitomir, Erika Nassar, Julie Culbertson, Jen Moreillon, Thomas Buford, Geoffrey Hudson, Matt Cooke, Richard Kreider, Darryn S Willoughby

**Affiliations:** 1 Department of Health, Human Performance, and Recreation, Baylor University, Box 97313, Waco, TX 76798, USA; 2 Institute for Biomedical Science, Baylor University, Waco, TX 87898, USA; 3 Department of Health and Kinesiology, Texas A&M University, College Station, TX 78743, USA

## Abstract

The purpose of this study was to evaluate the effects of a thermogenic supplement, Meltdown, on energy expenditure, fat oxidation, and hemodynamics before and after maximal treadmill exercise. In a double-blind, randomized, placebo-controlled, cross-over design, 12 male participants underwent two testing sessions after consuming either the Meltdown or placebo supplement. While in a fasted state, participants rested for one hour, orally ingested either Meltdown or placebo and rested for another hour, performed a maximal treadmill exercise test, and then rested for another hour. Throughout the testing protocol, resting energy expenditure (REE) and respiratory exchange ratio (RER) were assessed. In addition, heart rate (HR) and blood pressure (BP) were assessed before and after exercise. Meltdown increased REE significantly more than placebo at 45 min (1.44 ± 0.25 vs. 1.28 ± 0.23 kcal/min; p = 0.003), 60 min (1.49 ± 0.28 vs. 1.30 ± 0.22 kcal/min; p = 0.025), and 120 min (1.51 ± 0.26 vs. 1.33 ± 0.27 kcals/min; p = 0.014) post-ingestion. Meltdown significantly decreased RER at 30 min (0.84 ± 0.03 vs. 0.91 ± 0.04; p = 0.022) and 45 min post-ingestion (0.82 ± 0.04 vs. 0.89 ± 0.05; p = 0.042), and immediately post-exercise (0.83 ± 0.05 vs. 0.90 ± 0.07; p = 0.009). Furthermore, over the course of the evaluation period, area under the curve assessment demonstrated that REE was significantly increased with Meltdown compared to placebo (992.5 ± 133.1 vs. 895.1 ± 296.1 kcals; p = 0.043), while RER was significantly less than placebo (5.55 ± 0.61 vs. 5.89 ± 0.44; p = 0.002) following ingestion. HR and BP were not significantly affected prior to exercise with either supplement (p > 0.05) and the exercise-induced increases for HR and BP decreased into recovery and were not different between supplements (p > 0.05). These data suggest that Meltdown enhances REE and fat oxidation more than placebo for several hours after ingestion in fully rested and post-exercise states without any adverse hemodynamic responses associated with maximal exercise.

## Background

Despite a restructured food guide pyramid, ambitious Healthy People 2010 guidelines, and an explosion of weight loss products, there is an absence of systemic obesity relief. In fact, obesity is a growing concern and the problem may be accelerating. According to the CDC and results from the Behavioral Risk Factor Surveillance System (BRFSS) survey, obesity rates grew from 19.8% in 2000 to 23.9% in 2005, which is an increase of about 0.82% per year; obesity grew from 23.9% in 2005 to 25.6% in 2007, representing a 0.9% yearly increase [1]. The ramifications of the escalating obesity epidemic include an increased incidence of chronic disease and annual health care expenditure, as well as lost productivity [2].

Consumers often ingest diet products to bolster weight loss efforts. Formerly, ephedrine and ephedra were integral components in weight loss supplements. Since the most substantial effect of ephedrine use is enhanced sympathetic nervous system (SNS) activity via increased norepinephrine (NE) release from the nerve terminal, the drug has an impact on all adrenergic receptors (AR). Moreover, the ephedrine compound also has β-AR agonist and α-AR antagonist activity in circulation [3]. Previous investigations indicate that supplemental weight loss products containing a combination of ephedra (*ma huang*) or ephedrine and caffeine increase resting energy expenditure (REE) and loss of fat mass [4-6]. Therefore, prudent supplementation with ephedra-containing products, in combination with a sensible diet and exercise plan, has the potential to accelerate weight loss.

On April 12, 2004, however, ephedra-containing products were condemned by the FDA in response to safety concerns; therefore, manufacturers of nutrition supplements sought to formulate alternative thermogenic weight loss products with comparable effectiveness. As such, supplements containing caffeine and β-agonist compounds similar to ephedra began to appear on the market. Therefore, the *Citrus aurantium*-derived alkaloids synephrine and octapamine have gained popularity. Meltdown is a thermogenic product that combines synephrine, caffeine, phenylethylamine, yohimbine, hordenine and other compounds to stimulate the β-AR, thereby activating cyclic AMP (cAMP), while also inhibiting the α-AR that illicit an inhibitory response.

Activation of the SNS neurons stimulates the release of two primary catecholamine signaling molecules, epinephrine (E) and NE. After NE acts as an SNS stimulatory neurotransmitter (NT) in the brain, the adrenal medulla is prompted to release NE into blood. E or NE binding to an AR will produce a range of responses, the nature of the response depends upon on the receptor subtype to which the hormone binds [7].

Adrenergic analogues utilized in a thermogenic supplement are likely to enhance the activation of the SNS. For instance, the compound synephrine, which structurally resembles ephedrine, is primarily a sympathomimetic α1-AR agonist [8]. Typically, activation of the α1-AR mainly results in smooth muscle contraction, which causes vasoconstriction in blood vessels of the skin, GI tract, kidney and brain. Furthermore, one study revealed that synephrine also has moderate non-selective β3-AR agonist activity in several cell lines [9]. As previously revealed by Weyer et al. [10], supplementation with a selective β3-AR agonist, specifically CL 316,243, stimulates lipolysis in healthy men. Therefore, if synephrine is a sufficiently potent β3-AR agonist, the compound may promote fat and weight loss in humans through enhanced lipolysis in adipose tissue.

Caffeine is also a popular addition to thermogenic compounds, and two of the effects of caffeine are particularly relevant. Specifically, caffeine serves as an antagonist to adenosine in the neurons, via competitive inhibition, which stimulates the SNS [11]. Therefore, NE, dopamine and GABA secretion are increased in the brain, which prompts a greater release of catecholamines into the systemic circulation. Furthermore, since caffeine is also a known non-selective inhibitor of the enzyme cAMP-phosphodiesterase (PDE), which converts cAMP to its inactive, non-cyclic form, the compound amplifies the signal of cAMP through inhibition of PDE [12]. By promoting high concentrations of cAMP, caffeine intensifies and prolongs the effects of E and NE, as well as other stimulatory AR agonists, such as synephrine and octopamine. The thermogenic additive phenylethylamine also inhibits the reuptake of NE from the synaptic cleft [13]. Finally, yohimbine acts as a α2-AR antagonist [14]. Since α2-AR exist on the adrenal medulla as a negative feedback system for catecholamine release, a α2-AR antagonist may be of benefit in a weight loss supplement as a permissive promoter of NE and E release and signaling [15]. Finally, hordenine, like ephedrine, may have non-specific effects on AR by stimulating the release of NE [16], though human data is lacking.

When these compounds are combined in supplement form, they may work synergistically to enhance weight loss. Initially, phenylethylamine, caffeine, and hordenine may stimulate the SNS in a non-specific manner. Once SNS stimulation is initiated, N and NE circulate in higher than normal amounts, which activate AR throughout the body. Simultaneously, the inhibitory effects of the α_2_-AR are blocked by yohimbine. Furthermore, stimulatory SNS responses are enhanced by synephrine, which promotes the activity of Gs and subsequent cAMP signaling cascades. Finally, caffeine potentiates all cAMP activity by specifically inhibiting PDE.

The purpose of this study was to determine the effects of a single dose of the thermogenic supplement, Meltdown, on markers of hemodynamic function and energy expenditure at rest and during recovery from intense cardiovascular exercise. Therefore, the specific aims of this study were to assess the effects of Meltdown on: 1) REE for one hour prior to and one hour following a single bout of intense treadmill exercise and 2) hemodynamic and metabolic parameters during a single bout of intense treadmill exercise.

## Methodology

### Participants

Twelve apparently healthy and recreationally active male volunteers (23.67 +/- 4.66 yr, 175.05 +/- 7.16 cm, 79.89 +/- 18.01 kg) who were mild caffeine users (approximately 80 mg caffeine/day) participated in the study. All participants were cleared for participation by passing a mandatory medical screening. Participants with contraindications to exercise as outlined by the American College of Sports Medicine or who had consumed any nutritional supplements (excluding multi-vitamins) for two months prior to the study were not allowed to participate. All participants provided oral and informed written consent based on university-approved documents approved by the Institutional Review Board for Human Subjects for the Protection of Humans in Research. Additionally, all experimental procedures conformed to the ethical guidelines of the Helsinki Code. The purpose of the research and experimental procedures were explained to all study participants.

### Experimental protocol

In a double-blind, placebo-controlled, cross-over design, participants participated in two testing sessions after consuming either the Meltdown or placebo supplement. At each testing session, participants rested during a baseline assessment of REE for one hour, during which time respiratory exchange ratio (RER) was also determined as an indicator of macronutrient oxidation. At the conclusion of the resting/baseline measurements, participants orally ingested 3 capsules (500 mg) of Meltdown (Vital Pharmaceuticals, Davie, FL) or 3 capsules of cellulose placebo (500 mg) based on random assignment. Specifically, three capsules of Meltdown contains the following: 317 mg of a proprietary blend of caffeine anhydrous, α-methyl tetradecylthioacetic acid, yerba mate extract, and cAMP; 20 mg of methyl-synephrine HCl, 138 mg of a proprietary blend of β-methylphenylethylamine and methyl-β-phenylethylamine; 9 mg of a proprietary blend of 11-hydroxy yohimbine, yohimbine HCl, and α-yohimbine; 20 mg of methyl-hordenine HCl. Upon ingesting the supplement, the participants rested during another hour of REE measurement, during which time REE and RER were assessed every 15 min. At this point, participants performed a maximal aerobic treadmill test employing the Bruce protocol to assess their VO_2max _values using an open circuit spirometry system (Parvo Medics, Sandy, UT); HR and BP was assessed before and immediately following the exercise test. Upon completion of the exercise test, participants entered a one-hour recovery period in which REE and RER, were determined every 15 min.

### Dietary records

The participants' diets were not standardized and were asked not to change their dietary habits during the course of the study. However, subjects were required to keep dietary records for 48 hours prior to each testing session. The 48-hour dietary records were evaluated with the Food Processor dietary assessment software program to determine the average daily macronutrient consumption of fat, carbohydrate, and protein in the diet prior to supplementation and exercise.

### Resting energy expenditure test

Prior to and following exercise, participants rested supine on a padded table. Throughout the test, the participants had their expired gases monitored continuously for one hour throughout the test to determine REE and RER as an estimate of substrate oxidation using the using the 2400 TrueMax Metabolic Measurement System (Parvo Medics, Sandy, Utah).

### Assessment of Hemodynamic Variables (Heart Rate & Blood Pressure)

Prior to, during, and following exercise, participants' hemodynamic safety markers (HR and BP) were monitored. Heart rate was determined by a Polar heart rate monitor, and blood pressure was assessed in the supine position with a mercurial sphygmomanometer using standard procedures.

### Aerobic exercise capacity test

Participants completed maximal exercise on a treadmill (Quinton Q-Stress TM65, Bothell, WA) utilizing the Bruce protocol. The participants breathed through a sanitized mouthpiece attached to a head harness and a nose-clip. Throughout the test, expired gases were collected using the 2400 TrueMax Metabolic Measurement System (Parvo Medics, Sandy, Utah) during the warm-up and throughout exercise to exhaustion. During exercise, HR, BP, RER, and VO_2max _were determined.

### Statistical analyses

Statistical analyses were performed by utilizing separate 2 × 6 [Supplement (Meltdown, placebo] × Test (pre-rest, post-rest, pre-exercise, post, exercise, pre-recovery, post-recovery) factorial analyses of variance (ANOVA) with repeated measures for each criterion variable. Significant between-group differences were determined involving the Neuman-Keuls Post Hoc Test. However, to protect against Type I error, the conservative Hunyh-Feldt Epsilon correction factor was used to evaluate observed within-group F-ratios. In addition, area under the curve (AUC) was calculated by integral calculus for REE and RER. A Student's paired t-test was performed on the AUC for REE and RER and also for VO_2max_. All statistical procedures were performed using SPSS 11.0 software and a probability level of < 0.05 was adopted throughout.

## Results

Hemodynamic variables (HR and BP) were not significantly affected prior to exercise with either supplement (p > 0.05) and the expected exercise-induced increases observed in HR and BP that decreased into recovery were not different between supplements (p > 0.05). Relative to any supplement-induced differences in exercise performance, VO_2max _assessed at each of the testing sessions demonstrated no significant differences between Meltdown and placebo (45.31 ± 6.10 vs. 41.69 ± 9.98 ml O_2_/kg/min, p = 0.185).

**Figure 1:**
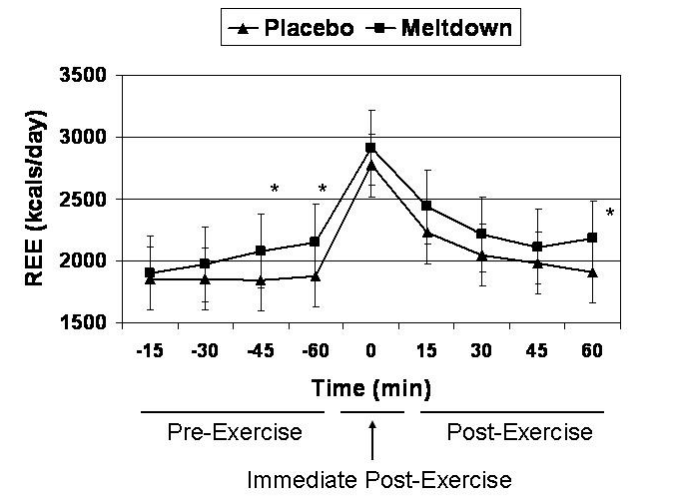
**An illustration of the effects of Meltdown and placebo on REE over the course of the evaluation period.** * indicates that Meltdown significantly elevated REE at 45 min (p = 0.003) and 60 min post-ingestion (p = 0.025), and at 60 min post-exercise (p = 0.014). Data are presented as means ± SD.

**Figure 2:**
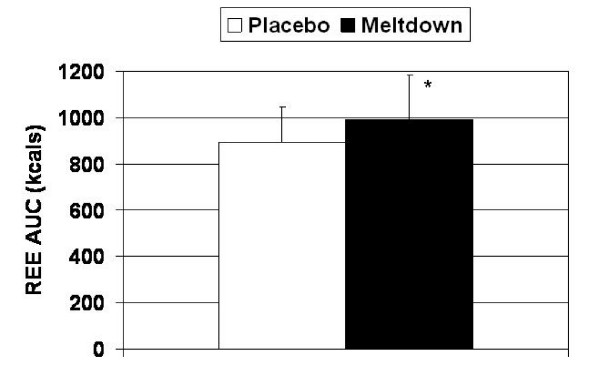
**Area under the curve analysis demonstrated that Meltdown significantly increased REE compared to placebo (p = 0.043).** Data are presented as means ± SD.

Meltdown increased REE significantly more than placebo at 45 min (1.44 ± 0.25 vs. 1.28 ± 0.23 kcal/min; p = 0.003) and 60 min (1.49 ± 0.28 vs. 1.30 ± 0.22 kcal/min; p = 0.025) post-ingestion. Furthermore, REE 60 min post-exercise (120 min following supplement administration) was significantly higher in the Meltdown group (1.51 ± 0.26 vs. 1.33 ± 0.27 kcals/min; p = 0.014) (Figure 1). Over the course of the evaluation period, AUC analysis demonstrated that REE was significantly increased with Meltdown compared to placebo (992.5 ± 133.1 vs. 895.1 ± 296.1 kcals; p = 0.043) (Figure 2).

**Figure 3:**
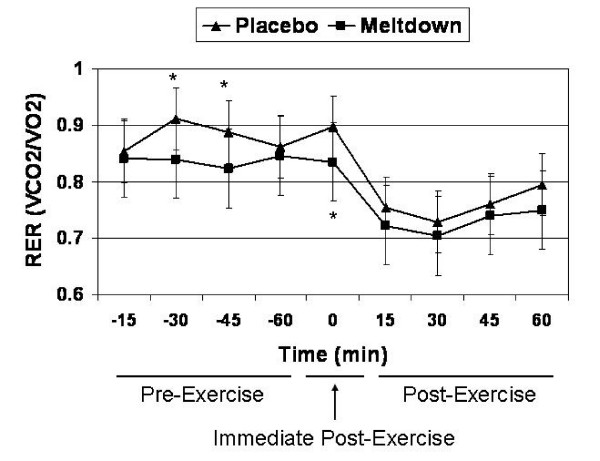
**An illustration of the effects of Meltdown and placebo on RER over the course of the evaluation period.** * indicates that Meltdown significantly decreased RER at 30 min (p = 0.022) and 45 min post-ingestion (p = 0.042), as well as immediately post-exercise (p = 0.009). Data are presented as means ± SD.

**Figure 4:**
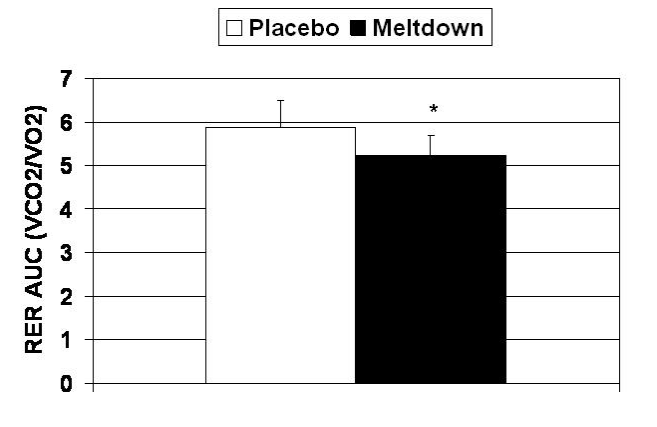
**Area under the curve analysis demonstrated that Meltdown significantly decreased RER compared to placebo (p = 0.002).** Data are presented as means ± SD.

Meltdown significantly decreased RER, compared to placebo, at 30 min (0.84 ± 0.03 vs. 0.91 ± 0.04; p = 0.022) and 45 min post-ingestion (0.82 ± 0.04 vs. 0.89 ± 0.05; p = 0.042), and immediately post-exercise (0.83 ± 0.05 vs. 0.90 ± 0.07; p = 0.009) (Figure 3). Furthermore, over the course of the evaluation period, AUC analysis demonstrated that RER significantly decreased with Meltdown compared to placebo (5.55 ± 0.61 vs. 5.89 ± 0.44; p = 0.002) (Figure 4).

## Discussion

The purpose of the present study was to determine the effects of Meltdown, a commercially available thermogenic nutritional supplement, on HR, BP, REE, and RER before, during, and after exercise. The results of this study show that Meltdown supplementation significantly elevated REE up to 120 min post-ingestion, while also decreasing RER up to 60 min post-ingestion. However, no significant differences were observed between Meltdown and placebo after a bout of maximal treadmill exercise, which was conducted 75 minutes after supplementation. In addition, no significant differences between Meltdown and placebo were observed for HR or BP before, during, or after exercise. Therefore, the symathomimetic compounds within Meltdown were not associated with any differential effects on hemodynamic function during exercise.

Thermogenic supplements may increase REE and fat metabolism through activation of the β2 and β3-AR. β2-AR stimulation has also been shown to up-regulate uncoupling proteins in skeletal muscle, which may have a significant impact on whole body thermogenesis [17,18]. Furthermore, the β3-AR, located primarily on adipocytes, enhances lipolysis and thermogenesis. It has been determined that beta-AR agonists with high thermogenic, antiobesity, and antidiabetic activities are potent stimulators of the β3-AR [19]. Given that the stimulation of the β3-AR primarily promotes lipolysis, it appears to be a good target for weight management and obesity treatment.

Previous studies examining the efficacy and safety of several alleged thermogenic compounds such as caffeine, citrus aurantium, garcinia cambogia, green tea extract, and ephedra have been conducted [5,6,12,20-24]. Moreover, studies involving ephedra or ephedra/caffeine revealed that that these compounds, especially in combination, may have significant potential to increase daily energy expenditure. In fact, the ephedrine/caffeine combination is reported to increase energy use 37.4–114 kcal/day more than placebo [21-24]. For example, one study investigated the changes in REE for three hours following the administration of 150 mg of caffeine and herbal ephedra containing 20 mg of ephedrine alkaloids [20]. During the final 30 min of measurement, REE was 8.5 ± 2.0% higher (P < 0.05) in the caffeine/ephedra trial compared to the placebo trial. A significant increase in REE was observed for caffeine/ephedra 180 min post-ingestion (1.47 ± 0.10 vs. 1.34 ± 0.08 kcal/min). In the present study, Meltdown supplementation, as compared to placebo, induced an increase in REE at 60 min post-supplementation (1.49 ± 0.28 vs. 1.30 ± 0.22 kcal/min) comparable to the previously-mentioned ephedra/caffeine study [20]. Since Meltdown contains compounds with β-agonist activity, in comparison to previous studies [21-24] Meltdown may generate REE increases comparable to ephedrine, even though in the present study the increases were observed to occur approximately 120 minutes earlier.

Furthermore, another study revealed that Java Fit, a combination herb and coffee supplement product, containing green tea extract, garcinia cambogia and niacin, produced a 14.4% increase (1,858.23 ± 412.89 vs. 2179.75 ± 424.34 kcal/day or 1.29 ± 0.29 vs. 1.51 ± 0.29 kcal/min) over the baseline resting energy expenditure value three hours after administration in physically active adults [25]. This difference was significantly more than a 5.7% increase observed with a commercially available caffeine-containing coffee. On the other hand, no significant changes were observed in RER during this study, and no significant changes in HR or BP were observed for either coffee product. In addition, another study conducted with Java Fit coffee concluded that this product has no negative effect on hemodynamic function and may increase REE in individuals who are sensitive to caffeine and herbs contained within the coffee supplement [26]. Therefore, the results of these two studies indicate that a mixed herbal product containing caffeine, green tea extract, garcinia cambogia and niacin induced a significant increase in energy expenditure without a simultaneous increase in hemodynamic safety variables in a young, fit population.

In addition to overall energy use, the use of fat as a source of energy, as indicated by an RER close to 0.7, is also of interest in metabolic studies. Furthermore, greater use of fat as an energy source is also presumed to be favorable. In fact, a study of 775 healthy, non-obese young men revealed that a high fasting RER (> 0.86) is a predictor of weight gain in men with an initial BMI < 25 [27]. Therefore, a product that lowers RER may be beneficial for lean men. Respiratory exchange ratio was significantly decreased with Meltdown, as determined by AUC analysis. Since REE increased and RER decreased, the data suggest that the Meltdown supplement increased energy expenditure with a reliance on fat as the preferential source of substrate oxidation.

Though ephedra and caffeine combination products increase metabolic activity [21-24], products of this kind are also associated with increases in blood pressure and heart rate. For example, one study of 15 healthy, young (aged 26.7 ± 2.52 years) male and female participants revealed that a single dose of a Metabolife 356, which included 12 mg of ephedra and 40 mg of caffeine, resulted in increases in the mean maximal QTc interval and systolic BP. As such, there is concern that combination products designed to mimic the effects of the ephedra and caffeine will also cause significant increases in HR and BP [28]. Such an increase in HR and BP was observed in a study comparing placebo to Xenadrine EFX, a 19-component combination product containing both caffeine and synephrine [29]. Therefore, the concern that so-called ephedra-free thermogenic products may increase BP appears to be legitimate; however, the results of the present study do not suggest that Meltdown increases hemodynamic safety markers. On the other hand, the present study was conducted in apparently healthy, lean young men; therefore, the results may not translate to obese or morbid populations.

On the other hand, the individual components used in thermogenic supplement formulation often do not have significant effects on BP or HR. For instance, a previous study also showed that a 46.9 mg dose of synephrine alone does not raise BP or HR more than placebo in healthy adults [29]. Though caffeine is thought to have a pressor effect, as evidenced by some reviews and meta-analysis [30,31], previous research indicates that caffeine alone may not increase the HR or BP of healthy, young, non-obese participants aged 21–26 [31]. Specifically, during one investigation 11 male and female participants ingested a caffeine dose of 5 mg/kg body weight or placebo [32]. The caffeine dose caused notable side effects and a significant inotropic effect (as assessed by echocardiogram); however, no increases in HR or BP were observed. On the other hand, another study of 10 healthy men aged 21–39 showed that a bolus dose of 0.125 mg/kg dose of yohimbine increased BP significantly [33]; however, HR was not affected. In the present study, the Meltdown supplement did not significantly elevate HR and BP before, during, or after exercise, which suggests that ingestion of this thermogenic supplement does not appear to adversely affect hemodynamic responses.

In conclusion, these data presented herein suggest that Meltdown enhances fat oxidation and REE more than placebo for up to 60 min and 120 min, respectively, after ingestion in fully rested and post-exercise states without any adverse hemodynamic responses when administered to apparently healthy young men.

## Competing interests

The authors declare that they have no competing interests.

## Authors' contributions

JJ participated in the design of the study, coordination and data acquisition, and assisted in performing the statistical analysis and drafting the manuscript. EN, JC, JM, TB, and GH participated in the data acquisition. MC assisted in graduate student supervision and participated data acquisition. RK assisted with providing graduate student funding and laboratory availability. DSW conceived the study, developed the study design, secured the funding for the project, assisted and provided oversight for all data acquisition and statistical analysis, assisted in drafting the manuscript, and served as the faculty mentor for the project. All authors have read and approved the final manuscript.
